# Entropy Generation via Ohmic Heating and Hall Current in Peristaltically-Flowing Carreau Fluid

**DOI:** 10.3390/e21050529

**Published:** 2019-05-24

**Authors:** Saima Noreen, Asif Abbas, Abid Hussanan

**Affiliations:** 1 Department of Mathematics, Comsats University Islamabad, Tarlai Kalan Park Road, Islamabad 44000, Pakistan; 2 Division of Computational Mathematics and Engineering, Institute for Computational Science, Ton Duc Thang University, Ho Chi Minh City 700000, Vietnam; 3 Faculty of Mathematics and Statistics, Ton Duc Thang University, Ho Chi Minh City 700000, Vietnam

**Keywords:** entropy, peristalsis, Carreau fluid, heat transfer, perturbation, pumping and trapping

## Abstract

The core objective of the present study is to examine entropy generation minimization via Hall current and Ohmic heating. Carreau fluid considerations interpret the unavailability of systems’ thermal energy (for mechanical work). The magneto hydrodynamic flow is in the channel, which is not symmetric. We have solved analytically the resulting nonlinear mathematical model. Moreover, physical exploration of important parameters on total entropy generation, temperature, and Bejan number is plotted and discussed. We observed that the generation of entropy takes place throughout the confined flow field *y* = *W*_1_ and *y* = *W*_2_ because of the viscous dissipation effect. In addition, reducing the operating temperature minimizes the entropy.

## 1. Introduction

Currently, scientists have a major concern about finding a way to control the wastage of heat energy. In thermodynamics, entropy defines thermal irreversibility, often referred to as the destruction of useful energy. Production of entropy is associated with all real-life process. Entropy generation analysis is important in exploring the sources and location of irreversibilities, which are responsible for the destruction of useful energy. The losses in heat energy are mainly due to friction, compression and expansion, heat transfer, magnetic field, and chemical reactions. Minimizing the loss of heat and improving the efficiency of the thermal system are possible only through minimization of entropy generation. Therefore, it is extremely important to study entropy in all real process. Different techniques are being used to decrease the entropy generation, such as the reduction in size of chip components in a computer, cooling fans preventing overheating, porous media, and the heat exchanger.

The laws of thermodynamics define the transformation of energy. The quantity of energy in the heat transfer process is an important factor and is governed by the first law. Hayat et al. [1] studied the impact of the Cattaneo–Christov heat flux model in the flow of variable thermally-conductive fluid. Khan et al. [2] explained the homogeneous-heterogeneous reactions in Casson fluid flow. Most of the engineering problems concern with the quality of energy and the degree of degradation of energy. The second law of thermodynamics defines the decrease in the quality of energy, such as the reduction in the quality of energy measured as entropy. In order to minimize the entropy generation within the fluid flow problem, it is important to learn the distribution of entropy generation. Bejan [3] laid the foundation of entropy generation and analyzed its minimization. Afridi et al. [4] developed the analysis of heat and mass transfer in entropy generation. In another study, Afridi et al. [5] analyzed entropy in hydromagnetic boundary flow. Rashidi et al. [6] studied the entropy generation on peristaltic MHD (magnetohydrodynamic) blood flow. In another article, Rashidi and his coworker analyzed the entropy generation of third-grade fluid over a stretching sheet [7]. Entropy generation in the flow of nano fluids with silver and copper nanoparticles was studied by Hayat et al. [8]. Khan et al. in his studies [9,10] also explained the ways of entropy generation minimization. Recently, some research works [11,12,13,14,15,16] investigated the entropy production numerically by LBM (Lattice Boltzmann Method).

Flows affected by magnetic field have crucial applications in various fields. MRI (magnetic resonance imaging), MHD pumps, the petroleum industry, plasma physics, etc., are few of the many modern applications. Moreover, when the magnetic field is strong, the Hall effect cannot be neglected. The Hall current and magnetic field have a strong effect on flow and heat transmission characteristics. Applications include the Hall accelerator, MHD accelerator, power generator, electric transformer, and refrigeration coils and heating elements. Hall current and the magnetic field are also used in MRA (magnetic resonance angiography), which scans the images of veins and arteries in order to analyze abnormalities of blood vessels, specifically arteries of brain, neck, thoracic aorta, and renal arteries. Abbasi et al. [17] developed numerical analysis for the peristaltic flow in a curved channel. Bhatti and Rashidi [18] examined the mass and heat transfer through the Hall current and Joule heating effects in blood flow. Recently, Noreen et al. [19] studied the effects of Joule heating and ion slip. Hayat et al. [20] studied the MHD viscous fluid flow phenomenon in the rotating channel. Qasim and Noreen [21] discussed the Hall current and viscous dissipation effect of pseudoplastic fluid. Some supplementary studies pursuing the same direction can also be found in [22,23,24].

Owing to real-life applications, peristaltic fluid motion has received considerable attention in the last few years. Peristaltic flow is a transport phenomenon in which fluid is carried forward through contractions and expansions. Significant industrial applications include the peristaltic pump, roller pump, blood pump, sanitary and sterile transport, etc. Physiologically, movement of food in esophagus, urine in ureter, blood in arteries, bile in bile ducts, etc., are based on this mode. Latham [25] laid a strong foundation for theoretical development in this area. He first addressed the peristaltic flow of viscous fluid. Shapiro et al. [26] extended the generalized concept under certain assumptions on peristaltic pumping. Asghar et al. [27] examined the variable viscosity of a viscous fluid. Vajravelu et al. [28] studied the peristaltically-flowing Phan-Thien–Tanner fluid of porous media in an asymmetric channel. Numerous examinations dealing with peristaltic fluid flows under different assumptions and flow geometry were reported with experimental, numerical, and analytical approaches. Some remarkable decisive studies were mentioned in [29,30,31,32,33].

The non-linear relationship between stress and strain rate is explored by selecting the Carreau fluid model, where the viscosity is dependent on the shear rate. This model is a blend of the power law and Newtonian models, competent for describing the flow behavior of shear thickening, as well as shear thinning fluids. Noreen et al. [34] analyzed peristaltic transport of Carreau fluid in a curved channel. In another article Hayat et al. [35] studied it with different boundary conditions.

In present study, the non-linear relationship between stress and strain rate is explored by selecting the Carreau fluid model, where the viscosity is dependent on the shear rate. This model is a blend of the power law and Newtonian models, competent for describing the flow behavior of shear thickening, as well as shear thinning fluids. Noreen et al. [34] analyzed peristaltic transport of Carreau fluid in a curved channel. In another article, Hayat et al. [35] studied the same model with different boundary conditions.

No study is available in the literature that explores the entropy generation of Carreau fluid flow. We present the state-of-the-art present entropy analysis of flow augmented by Hall current and Joule heating. Flow is in a two-dimensional channel with convective boundaries. The study is organized into five sections:

## 2. Mathematical Model and Analysis

### 2.1. Flow Characteristics

**Figure 1:**
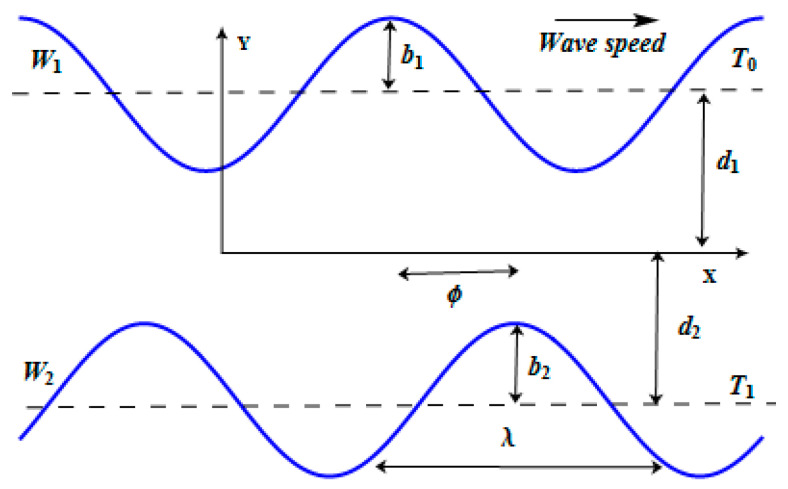
Physical model of the problem.

The Carreau fluid of constant density, moving in a channel, which is asymmetric in nature, is considered here. The rectangular coordinates are $\left(\bar{X},\;\bar{Y}\right)$ with U as the axial velocity component. Channel walls are maintained at temperatures $T_{0}$ and $T_{1}$. The velocity field is mathematically defined by $\left(\bar{U},\;\bar{V},\;0\right)$. The walls of the geometry are given as:

(1) $$\begin{array}{l} {\bar{W}}_{1}=b_{1}\cos\left(\frac{2\pi }{\lambda }\left(\bar{X}-s\bar{t}\right)\right)+d_{1}\\ {\bar{W}}_{2}=-b_{2}\cos\left(\frac{2\pi }{\lambda }\left(\bar{X}-s\bar{t}\right)+\phi \right)-d_{2} \end{array}$$

where ${\bar{W}}_{1}\;\mathrm{and}\;{\bar{W}}_{2}$ represent the lower and upper walls, $b_{1},\;b_{2}$ the amplitudes, ϕ the phase difference, s the wave speed, λ the wavelength, and $\bar{t}$ the time. The geometry of the flow problem is given in Figure 1.

The Lorentz body force and Joule heat affecting the flow are determined by:

(2) $$\bar{F}=\bar{J}\times \bar{B}$$

(3) $$\mathrm{Joule}\;\mathrm{heating}\;\mathrm{effect}=\frac{1}{\sigma }\bar{J}\cdot \bar{J}$$

Here, $\bar{J}$ shows the current density $\bar{B}$ for the magnetic field, and σ represents the electric conductivity of the fluid. The constitutive laws of mass, momentum, and energy via the Joule heating, Hall current, and viscous dissipation are:

(4) $$\frac{\bar{d\rho }}{\bar{dt}}+\nabla \cdot \bar{V}=0,$$

(5) $$\rho \frac{d\bar{V}}{d\bar{t}}=\mathrm{div}\;S-\bar{J}\times \bar{B,}$$

(6) $$\rho C_{p}\frac{d\bar{T}}{d\bar{t}}=\kappa \;\nabla^{2}\bar{T}+\bar{\tau }\cdot \left(grad\bar{V}\right)+\frac{1}{\sigma }\bar{J}\cdot \bar{J},$$

where $S=-pI+\bar{\tau ,}$ ρ shows density, κ is the thermal conductivity, $C_{p}$ the specific heat, and $\bar{\tau }$ the extra stress tensor.

### 2.2. Fluid Model

The stress-strain relationship of the Carreau fluid model is:

(7) $$\bar{\tau }=\left[\eta_{\infty }+\left(\eta_{\infty }-\eta_{0}\right){\left(1+{\left(\Gamma \dot{\gamma }\right)}^{2}\right)}^{\frac{n-1}{2}}\right]\bar{\dot{\gamma }},$$

and here, $\eta_{\infty },\;\eta_{0}$ are infinite and initial shear rate viscosities and $\bar{\dot{\gamma }}=\sqrt{\frac{1}{2}\mathrm{trace}A_{1}{}^{2}}$. The components of extra stress tensors ${\bar{\tau }}_{ij}$ are:

(8) $${\bar{\tau }}_{\bar{X}\bar{X}}=-2\eta_{0}\left[{\left(1+{\left(\Gamma \dot{\gamma }\right)}^{2}\right)}^{\frac{n-1}{2}}\right]\;\frac{\partial \bar{U}}{\partial \bar{X}},$$

(9) $${\bar{\tau }}_{\bar{X}\bar{Y}}=-\eta_{0}\left[{\left(1+{\left(\Gamma \dot{\gamma }\right)}^{2}\right)}^{\frac{n-1}{2}}\right]\;\left(\frac{\partial \bar{U}}{\partial \bar{Y}}+\frac{\partial \bar{V}}{\partial \bar{X}}\right),$$

(10) $${\bar{\tau }}_{\bar{Y}\bar{Y}}=-\eta_{0}\left[{\left(1+{\left(\Gamma \dot{\gamma }\right)}^{2}\right)}^{\frac{n-1}{2}}\right]\;2\frac{\partial \bar{V}}{\partial \bar{Y}}.$$

### 2.3. Development Problem

The transformation:

(11) $$\bar{x}=\bar{X}-c\bar{t},\;\bar{v}=\bar{V}\left(\bar{X},\bar{Y},\bar{t}\right),\;\bar{y}=\bar{Y},\;\bar{t}=\bar{T}\left(\bar{X},\bar{Y},\bar{t}\right),\;\bar{u}=\bar{U}\left(\bar{X},\bar{Y},\bar{t}\right)-c,$$

convert Equations (4)–(6) to:

(12) $$\frac{\partial \bar{u}}{\partial \bar{x}}+\frac{\partial \bar{v}}{\partial \bar{y}}=0$$

(13) $$\rho \left(\left(\bar{u}+c\right)\frac{\partial \bar{u}}{\partial \bar{x}}+\bar{v}\frac{\partial \bar{u}}{\partial \bar{y}}\right)=\frac{\partial {\bar{\tau }}_{\bar{x}\bar{x}}}{\partial \bar{x}}+\frac{\partial {\bar{\tau }}_{\bar{x}\bar{y}}}{\partial \bar{y}}-\frac{\sigma B_{0}{}^{2}}{1+m_{e}{}^{2}}\left(\left(\bar{u}+c\right)-m\bar{v}\right)-\frac{\partial \bar{p}}{\partial \bar{x}},$$

(14) $$\rho \left(\left(\bar{u}+c\right)\frac{\partial \bar{v}}{\partial \bar{x}}+\bar{v}\frac{\partial \bar{v}}{\partial \bar{y}}\right)=\frac{\partial {\bar{\tau }}_{\bar{x}\bar{y}}}{\partial \bar{x}}+\frac{\partial {\bar{\tau }}_{\bar{y}\bar{y}}}{\partial \bar{y}}-\frac{\sigma B_{0}{}^{2}}{1+m_{e}{}^{2}}\left(\bar{v}-m\left(\bar{u}+c\right)\right)-\frac{\partial \bar{p}}{\partial \bar{y}},$$

(15) $$\begin{array}{r} \rho {\;C}_{p}\left(\left(\bar{u}+c\right)\frac{\partial }{\partial \bar{x}}+\bar{v}\frac{\partial }{\partial \bar{y}}\right)\bar{T}=-\frac{\sigma B_{0}{}^{2}}{1+m_{e}{}^{2}}\left({\left(\bar{u}+c\right)}^{2}-{\bar{v}}^{2}\right)+\frac{\partial \bar{u}}{\partial \bar{x}}{\bar{\tau }}_{\bar{x}\bar{x}}+\frac{\partial \bar{v}}{\partial \bar{y}}{\bar{\tau }}_{\bar{x}\bar{y}}+\frac{\partial \bar{u}}{\partial \bar{x}}{\bar{\tau }}_{\bar{x}\bar{y}}+\frac{\partial \bar{v}}{\partial \bar{y}}{\bar{\tau }}_{\bar{y}\bar{y}}\\ +\kappa \left(\frac{\partial^{2}}{\partial {\bar{x}}^{2}}+\frac{\partial^{2}}{\partial {\bar{y}}^{2}}\right)\bar{T}. \end{array}$$

Introducing dimensionless variables and parameters:

(16) u=u¯c, x=x¯λ, t=ct¯λ, y=y¯d1, v=v¯cδ, Mf=σB02d12η0, p=d12p¯cλη0, τxx=λcη0τ¯x¯x¯, τxy=d1cη0τ¯x¯x¯, γ˙=γ˙¯d1c, τyy=d1cη0τ¯y¯y¯, θ=T¯−T0T1−T0, Pr=μCpκ, Ec=c2(T1−T0)CpW1=W¯1d1, We=Γcd1, W2=W¯2d2, δ=d1λ, Re=ρcd1η0,u=∂ψ∂y,v=−∂ψ∂x.

Equations (12)–(15), along with low Reynold number and long wavelength assumptions, yield:

(17) $$\frac{\partial p}{\partial x}=\frac{\partial }{\partial y}\left[1+\left(\frac{n-1}{2}\right)W_{e}{}^{2}{\left(\frac{\partial^{2}\psi }{\partial y^{2}}\right)}^{2}\right]\frac{\partial^{2}\psi }{\partial y^{2}}-\frac{M_{f}}{1+m_{e}{}^{2}}\left(1+\frac{\partial \psi }{\partial y}\right),$$

(18) $$\frac{\partial p}{\partial y}=0,$$

(19) $$\frac{\partial^{2}\theta }{\partial y^{2}}+B_{r}{\left(\frac{\partial^{2}\psi }{\partial y^{2}}\right)}^{2}\left[1+\left(\frac{n-1}{2}\right)W_{e}{}^{2}{\left(\frac{\partial^{2}\psi }{\partial y^{2}}\right)}^{2}\right]-\frac{M_{f}}{1+m_{e}{}^{2}}{\left(1+\frac{\partial \psi }{\partial y}\right)}^{2}=0$$

As p≠p(y), therefore Equation (17) yields:

(20) $$\left[1+\frac{3(n-1)}{2}W_{e}{}^{2}{\left(\frac{\partial^{2}\psi }{\partial y^{2}}\right)}^{2}\right]\frac{\partial^{4}\psi }{\partial y^{4}}+3(n-1)W_{e}{}^{2}{\left(\frac{\partial^{2}\psi }{\partial y^{2}}\right)}^{2}{\left(\frac{\partial^{3}\psi }{\partial y^{3}}\right)}^{2}-\frac{M_{f}}{1+m_{e}{}^{2}}\left(1+\frac{\partial \psi }{\partial y}\right)=0,$$

In the above equations, $B_{r},\;W_{e},\;M_{f},\;m_{e},\;\psi ,\;\mathrm{and}\;\theta$ are the notations for the Brinkman number, Weissenberg number, Hartman number, Hall parameter, stream function, and temperature, respectively. The associated non-dimensional boundary conditions are:

(21) $$\psi =-\frac{F}{2},\frac{\partial \psi }{\partial y}=-1,\frac{\partial \theta }{\partial y}+Bi_{1}(\theta -1)=0,\;\mathrm{at}\;y=W_{1},\;\psi =\frac{F}{2},\frac{\partial \psi }{\partial y}=-1,\frac{\partial \theta }{\partial y}+Bi_{2}\theta =0,\;\mathrm{at}\;y=W_{2},$$

where $F=\int_{W_{1}}^{W_{2}}\frac{\partial \psi }{\partial y}dy$ is related to the fixed frame by F=Q−1−d.

## 3. Analysis of Entropy Generation

Fluid irreversibilities in the current problem are due to heat diffusion, viscous dissipation, and the magnetic field, respectively. Based on these, the dimensional entropy generation is defined as:

(22) $${\bar{S}}_{G}=\frac{\kappa }{{\bar{T}}^{2}}\left[{\left(\frac{\partial T}{\partial \bar{X}}\right)}^{2}+{\left(\frac{\partial T}{\partial \bar{Y}}\right)}^{2}\right]+\frac{1}{\bar{T}}\left[\frac{\sigma B_{0}{}^{2}}{1+m_{e}{}^{2}}\left({\bar{U}}^{2}+\bar{V}\right)\right]+\frac{1}{\bar{T}}\left[\tau \cdot \left(grad\bar{V}\right)\right].$$

The characteristic entropy is defined as $S_{0}=\frac{d_{1}{}^{2}}{\kappa }.$ The total entropy generation rate, denoted by $N_{ts}$, is the relation between the actual entropy to the characteristic entropy. In dimensional form, entropy generation via stream function presentation:

(23) $$N_{ts}=\frac{S_{G}}{S_{0}},$$

(24) $$N_{ts}=\underset{N_{H}}{\underset{︸}{\frac{1}{{\left(\theta +\xi \right)}^{2}}{\left(\frac{\partial \theta }{\partial y}\right)}^{2}}}+\underset{N_{F}}{\underset{︸}{\frac{B_{r}}{\left(\theta +\xi \right)}\left[{\left(\frac{\partial^{2}\psi }{\partial y^{2}}\right)}^{2}+\left(\frac{n-1}{2}\right)W_{e}{}^{2}{\left(\frac{\partial^{2}\psi }{\partial y^{2}}\right)}^{4}\right]}}+\underset{N_{M}}{\underset{︸}{\frac{B_{r}}{\left(\theta +\xi \right)}\frac{M_{f}}{1+m_{e}{}^{2}}{\left(1+\frac{\partial \psi }{\partial y}\right)}^{2}}}$$

Here, the temperature difference parameter is labelled by $\xi =\frac{T_{0}}{T_{1}-T_{0}}$, and $N_{ts}=N_{H}+N_{F}+N_{M}$.

The Bejan number identified by $B_{e}$ is the proportion of heat irreducibility to the total entropy generation. Basically, the $B_{e}$ number comprehends the mechanism of the production of entropy.

(25) $$B_{e}=\frac{\frac{1}{{\left(\theta +\xi \right)}^{2}}{\left(\frac{\partial \theta }{\partial y}\right)}^{2}}{\frac{1}{{\left(\theta +\xi \right)}^{2}}{\left(\frac{\partial \theta }{\partial y}\right)}^{2}+\frac{B_{r}}{\left(\theta +\xi \right)}\left[{\left(\frac{\partial^{2}\psi }{\partial y^{2}}\right)}^{2}+\left(\frac{n-1}{2}\right)W_{e}{}^{2}{\left(\frac{\partial^{2}\psi }{\partial y^{2}}\right)}^{4}\right]+\frac{B_{r}}{\left(\theta +\xi \right)}\frac{M_{f}}{1+m_{e}{}^{2}}{\left(1+\frac{\partial \psi }{\partial y}\right)}^{2}}$$

$B_{e}=\frac{1}{2}$ defines equal irreversibility, due to heat and other contributing factors. For dominating heat irreversibilities, $B_{e}=1$, while $B_{e}=0$ implies that the contributing factors of fluid friction and magnetic field are noteworthy. The Bejan number ranges between zero and one.

## 4. Solution Methodology

Our problem is non-linear and coupled in nature. The computation of the exact solution is not possible; therefore, perturbation techniques are employed to solve the resulting governing equations. We apply regular perturbation of the fluid parameter, the Weissenberg number $W_{e}{}^{2}$ as:

(26) $$\psi =\psi_{0}+W_{e}{}^{2}\psi_{1}+O{\left(W_{e}{}^{2}\right)}^{2},\;p=p_{0}+W_{e}{}^{2}p_{1}+O{\left(W_{e}{}^{2}\right)}^{2},\;F=F_{0}+W_{e}{}^{2}F_{1}+O{\left(W_{e}{}^{2}\right)}^{2},\;\theta =\theta_{0}+W_{e}{}^{2}\theta_{1}+O{\left(W_{e}{}^{2}\right)}^{2}.$$

Substituting these into Equations (19) and (20), we construct the zeroth order and first order systems with reference to the fluid parameter.

### 4.1. Zeroth Order System and Boundary Conditions

(27) $$-\frac{M_{f}}{1+m_{e}{}^{2}}\left(\frac{\partial^{2}\psi_{0}}{\partial y^{2}}\right)+\left(\frac{\partial^{4}\psi_{0}}{\partial y^{4}}\right)=0$$

(28) $$\frac{M_{f}}{1+m_{e}{}^{2}}B_{r}+2B_{r}\frac{M_{f}}{1+m_{e}{}^{2}}\left(\frac{\partial \psi_{0}}{\partial y}\right)+B_{r}\frac{M_{f}}{1+m_{e}{}^{2}}{\left(\frac{\partial \psi_{0}}{\partial y}\right)}^{2}+\frac{\partial^{2}\theta_{0}}{\partial y^{2}}+B_{r}\left(\frac{\partial^{2}\theta_{0}}{\partial y^{2}}\right)=0$$

(29) $$\begin{array}{l} \psi_{0}=-\frac{F_{0}}{2},\frac{\partial \psi_{0}}{\partial y}=-1,\frac{\partial \theta_{0}}{\partial y}+Bi_{1}(\theta_{0}-1)=0,\;\mathrm{at}\;y=W_{1}\\ \psi_{0}=\frac{F_{0}}{2},\frac{\partial \psi_{0}}{\partial y}=-1,\frac{\partial \theta_{0}}{\partial y}+Bi_{2}\theta_{0}=0,\;\mathrm{at}\;y=W_{2} \end{array}$$

### 4.2. First-Order System and Boundary Conditions

(30) $$-\frac{M_{f}}{1+m_{e}{}^{2}}\left(\frac{\partial \psi_{1}}{\partial y}\right)+3(n-1)\left(\frac{\partial^{2}\psi_{0}}{\partial y^{2}}\right){\left(\frac{\partial^{3}\psi_{0}}{\partial y^{3}}\right)}^{2}+\frac{3}{2}(n-1)\left(\frac{\partial^{2}\psi_{0}}{\partial y^{2}}\right)\left(\frac{\partial^{4}\psi_{0}}{\partial y^{4}}\right)+\left(\frac{\partial^{4}\psi_{1}}{\partial y^{4}}\right)=0$$

(31) $$\begin{array}{l} 2B_{r}\frac{M_{f}}{1+m_{e}{}^{2}}\left(\frac{\partial \psi_{1}}{\partial y}\right)+2B_{r}\frac{M_{f}}{1+m_{e}{}^{2}}\left(\frac{\partial \psi_{0}}{\partial y}\right)\left(\frac{\partial \psi_{1}}{\partial y}\right)+\frac{\partial^{2}\theta_{1}}{\partial y^{2}}+\frac{1}{2}(n-1)B_{r}{\left(\frac{\partial^{2}\psi_{0}}{\partial y^{2}}\right)}^{4}\\ +2B_{r}{\left(\frac{\partial^{2}\psi_{0}}{\partial y^{2}}\right)}^{4}+2B_{r}\left(\frac{\partial^{2}\psi_{0}}{\partial y^{2}}\right)\left(\frac{\partial^{2}\psi_{1}}{\partial y^{2}}\right)=0 \end{array}$$

(32) $$\begin{array}{l} \psi_{1}=-\frac{F_{1}}{2},\frac{\partial \psi_{1}}{\partial y}=0,\frac{\partial \theta_{1}}{\partial y}+Bi_{1}\theta_{1}=0,\;\mathrm{at}\;y=W_{1}\\ \psi_{1}=\frac{F_{1}}{2},\frac{\partial \psi_{1}}{\partial y}=0,\frac{\partial \theta_{1}}{\partial y}+Bi_{2}\theta_{1}=0,\;\mathrm{at}\;y=W_{2} \end{array}$$

## 5. Discussion and Results

The impact of different physical parameters, i.e., $B_{r},\;W_{e},\;M_{f},\;m_{e},\;Bi_{1},\;Bi_{2},\;\mathrm{and}\;\phi$, on total entropy generation, entropy production due to the friction and heat diffusion, temperature, Bejan number, heat transfer rate, pressure gradient, streams lines, and velocity profile are discussed in this section.

### 5.1. Analysis of Entropy Generation $\left(N_{ts}\right)$ and Bejan Number $\left(B_{e}\right)$

**Figure 2:**
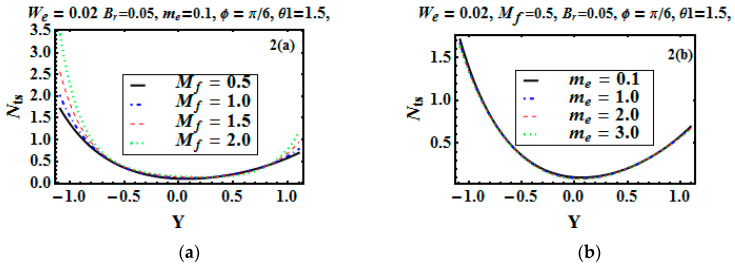

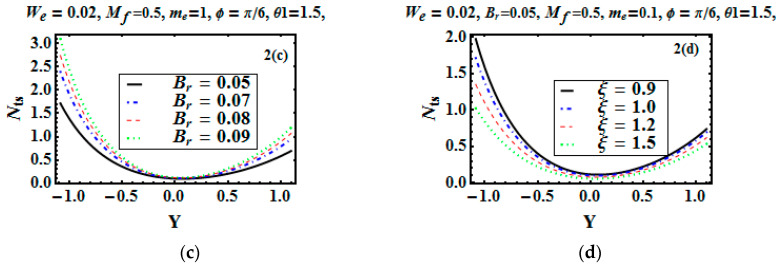
Entropy generation $N_{ts}$ versus *y* for different values of $M_{f},\;m_{e},\;B_{r}\;\mathrm{and}\;\xi$ (**a**–**d**).

**Figure 3:**
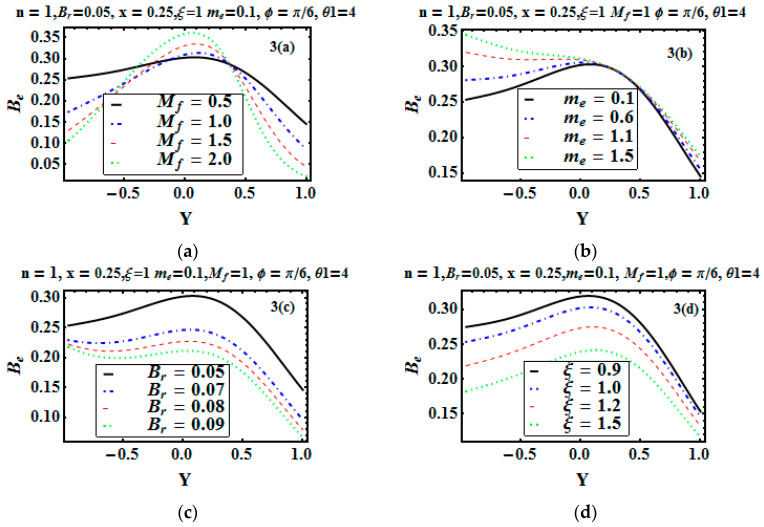
Bejan number $B_{e}$ versus y for various $M_{f},\;m_{e},\;B_{r}\;\mathrm{and}\;\xi$ (**a**–**d**).

The entropy generation $N_{ts}$ and Bejan number $B_{e}$ are plotted in Figure 2a–d and Figure 3a–d to depict the effects of the Hartman number, Hall parameter, Brinkman number, and temperature difference parameter. Figure 2a,c portrays that $N_{ts}$ was gradually enhancing for increasing values of the Hartman and Bejan numbers. With the application of the magnetic field, the temperature increased. Joule heating produced more heat, so entropy production increased. At the lower wall, entropy generation was maximum as compared to the other wall (due to temperature gradient). It can also be noticed that at the lower wall, fluid friction irreducibility was dominant, whereas at the upper wall, heat transfer reduced the entropy generation. Figure 2b shows the effect of the Hall parameter $m_{e}$, which reduced the entropy generation. In Figure 2c, as we increased the Brinkman number (the conduction of energy that was produced by viscous dissipation), entropy generation increased. Figure 2d depicts that with an increment in temperature difference, the entropy generation gradually decreased. Figure 3a indicates the Bejan number for the variation of the Hartman number. It reveals that heat irreversibility at the bulk fluid region was dominant, while at the edges, magnetic and viscous irreversibility were dominating. Figure 3b presents that Bejan number decreased with the Hall parameter $m_{e}$ at y=0. Figure 3c,d show that with the increase in the Brickman number and temperature difference parameter, the Bejan number decreased.

### 5.2. Analysis of Temperature

**Figure 4:**
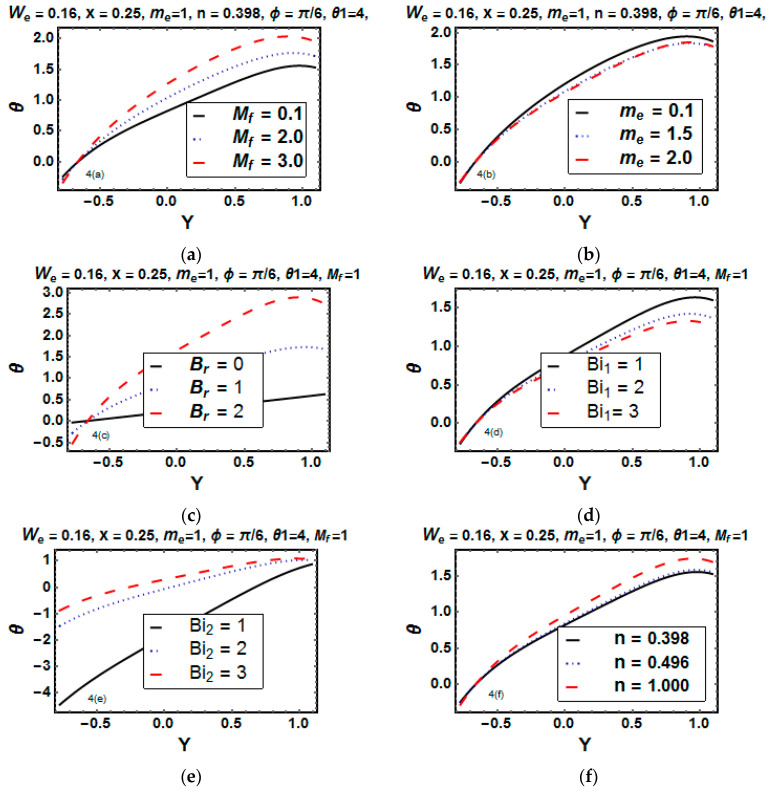
Temperature θ versus y for different values of $M_{f},\;m_{e},\;B_{r},\;Bi_{1},\;Bi_{2}\;\mathrm{and}\;n$ (**a**–**f**).

Figure 4a–f shows the behavior of temperature for different physical parameters, particularly the Hartman number $\left(M_{f}\right),$ Hall parameter $\left(m_{e}\right),$ Brinkman number $\left(B_{r}\right)$, power law index (*n*), and Biot numbers $\left(Bi_{1},\;Bi_{2}\right).$ Figure 4a depicts that the temperature profile gradually rose for increasing values of the Hartman number $M_{f}$. Basically, magnetic field lines interacted electrically with the fluid and produced Lorentz force. Lorentz force retarded the fluid motion (transforming the kinetic energy of the electrically conducting fluid to heat energy), and fluid temperature rose. It is found from Figure 4b that the temperature is lowered because of the increase in the electrical conductivity of the fluid. Figure 4c elucidates that under the influence of the Brinkman number, the temperature rose. The reason behind this is that for a large value of the Brickman number, the frictional force increased (due to the collision of fluid molecules with each other), and as a result, kinetic energy converted into thermal energy, implying a rise in total fluid temperature. The influence of the Biot number on the temperature is presented in Figure 4d,e. Temperature decreased at the upper wall by the increase of $Bi_{1}$, and it had no visible effect on the lower wall. In contrast, the temperature escalated at the lower wall with the increase of $Bi_{2}$, and a negligible difference was observed on the upper wall. In most of the cases, for small Biot numbers, temperature uniformly distributed inside the fluid, whereas for Biot numbers greater than 0.1, irregularity resulted. Therefore, we tool a special case for a large value of the Biot number. Figure 4f elucidates that temperature increased for increasing values of the power law index.

### 5.3. Analysis of Velocity

**Figure 5:**
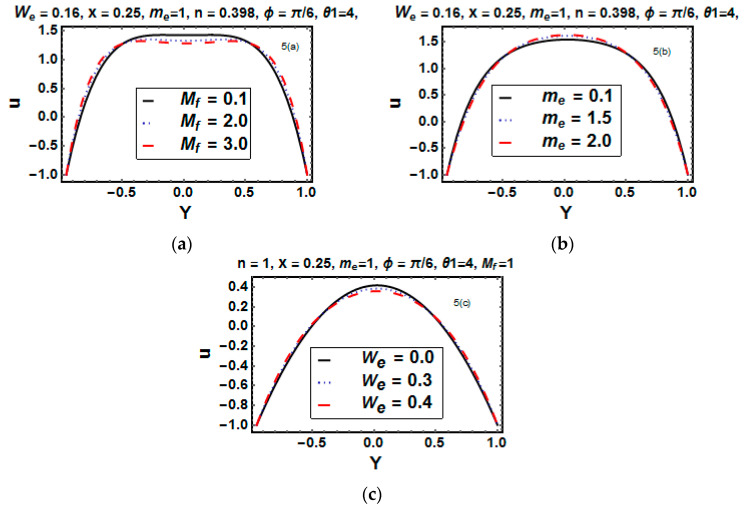
(**a**–**c**) Velocity u versus y for different values of $M_{f},\;m_{e}\;\mathrm{and}\;W_{e}$.

Axial velocity serves to provide salient feature of flow behavior. Figure 5a–c portrays the impact velocity profile in a channel with convective boundaries. We observed that the velocity formed a parabolic trajectory for physical parameters, and maximum velocity occurred at y=0. Figure 5a portrays that the axial velocity decreased for the increasing value of the Hartman number. Since the Hartman number directly relates the magnetic force and this force is resistive in nature, therefore the velocity decreased. Figure 5b demonstrates the influence of the Hall parameter $m_{e}$. Here, the velocity accelerated at the center of the channel while it reduced at the edges, the reasons behind this being that $m_{e}$ caused an upsurge of the electrical conductivity of the fluid; hence, the velocity increased. Figure 5c shows the comparison of viscous and Carreau fluids. It gained maximum velocity for a Newtonian fluid, while it reduced for the non-Newtonian Carreau fluid. Furthermore, it restored the symmetry about the center line.

### 5.4. Analysis of the Pressure Gradient and the Rate of Heat Transfer

**Figure 6:**
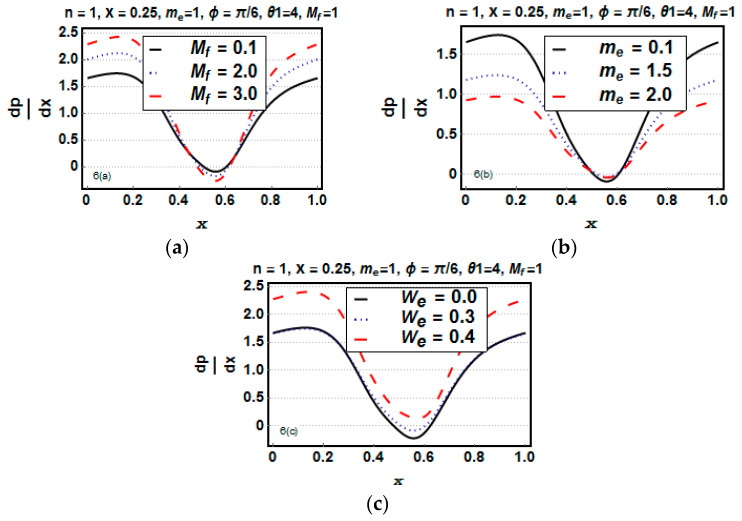
(**a**–**c**) Pressure gradient dp/dx versus *x* for different values of $M_{f},\;m_{e},\;\mathrm{and}\;W_{e}$.

**Figure 7:**
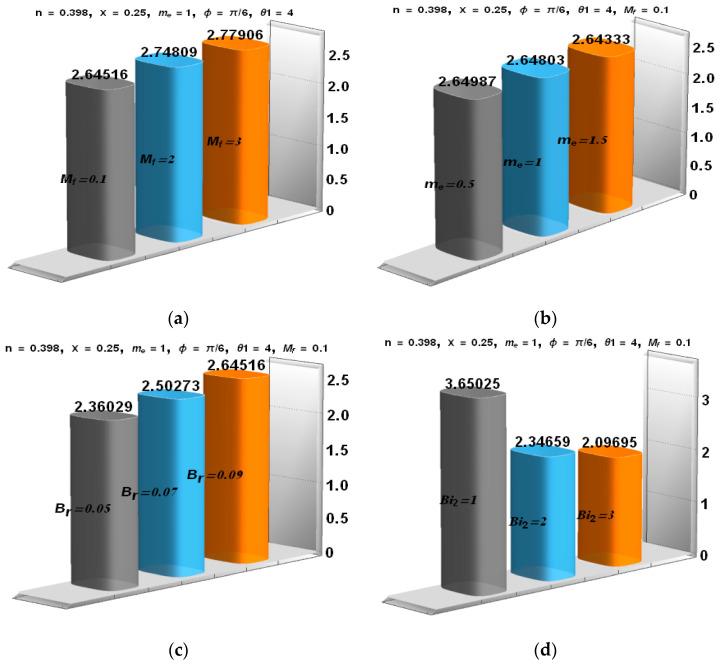
(**a–d**) Rate of heat transfer for different values of $M_{f},\;m_{e},\;B_{r}\;\mathrm{and}\;Bi_{2}$.

The influence of $M_{f},{\;m}_{e},\;W_{e},\;\mathrm{and}\;B_{r}$ is analyzed through Figure 6a–c and Figure 7a–d to peruse the pressure gradient and rate of heat transfer. Figure 6a reveals that for the increasing value of the Hartman number, the pressure gradient decreased at the narrow part, whereas it increased at the wider region. Figure 6b depicts the influence of the Hall parameter. dp/dx decreased at the wider region, and a negligible difference was observed at the narrow part. Figure 6c portrays that with the increase of the Weissenberg number, the pressure gradient dp/dx increased at the narrow and wider region. Figure 7a,d presents that for higher values of the Biot number and Hall parameter, the heat transfer rate reduced; whereas the heat transfer rate increased for the Brinkman number and Hartman number (Figure 7b,c).

### 5.5. Trapping Phenomenon

**Figure 8:**
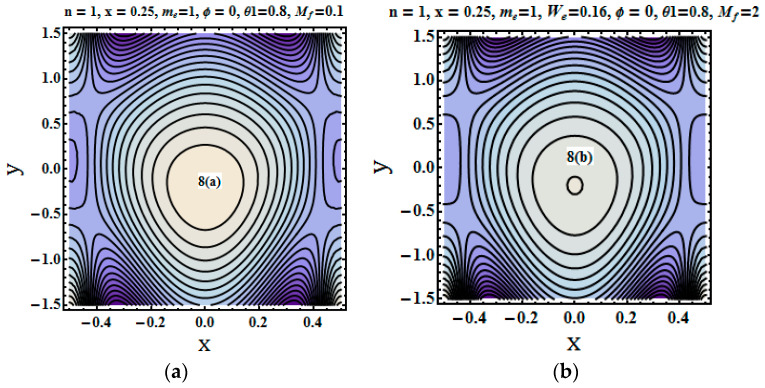

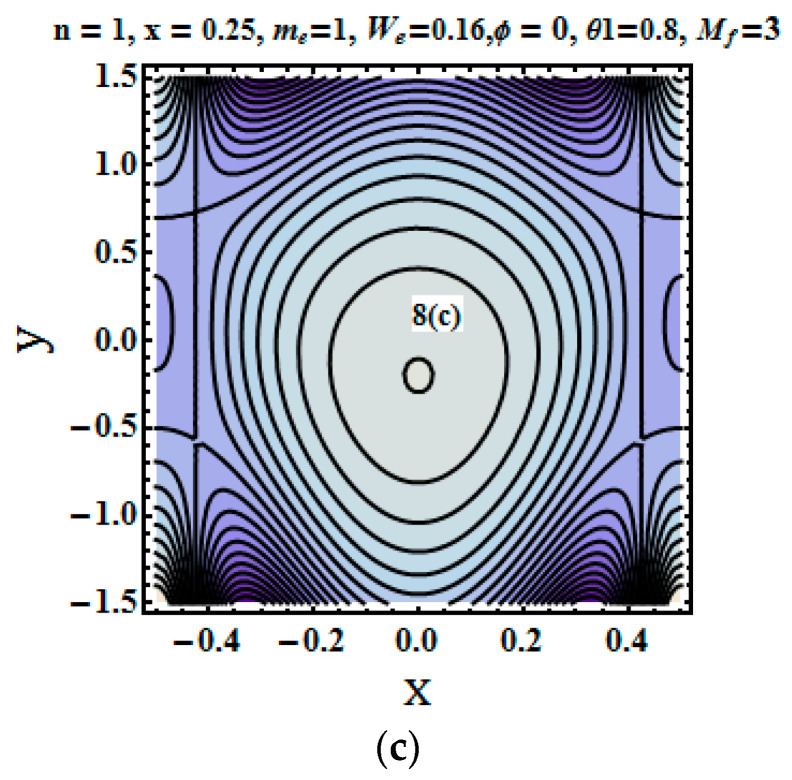
(**a**–**c**) Stream lines for different values of $M_{f}=0.1,\;M_{f}=2,\;M_{f}=3$.

**Figure 9:**
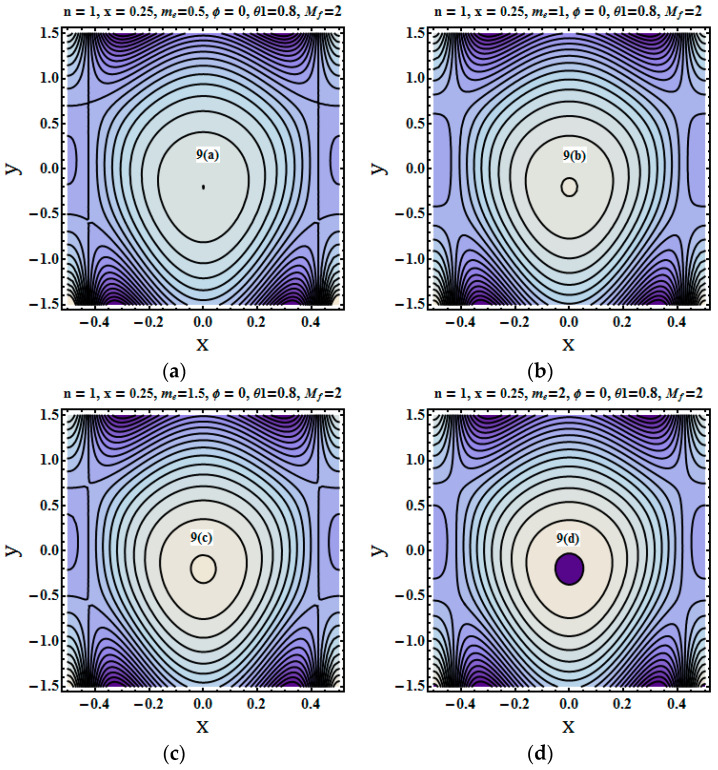
(**a**–**d**) Stream lines for different values of $m_{e}=0.5,\;m_{e}=1,\;m_{e}=1.5,\;m_{e}=2$.

**Figure 10:**
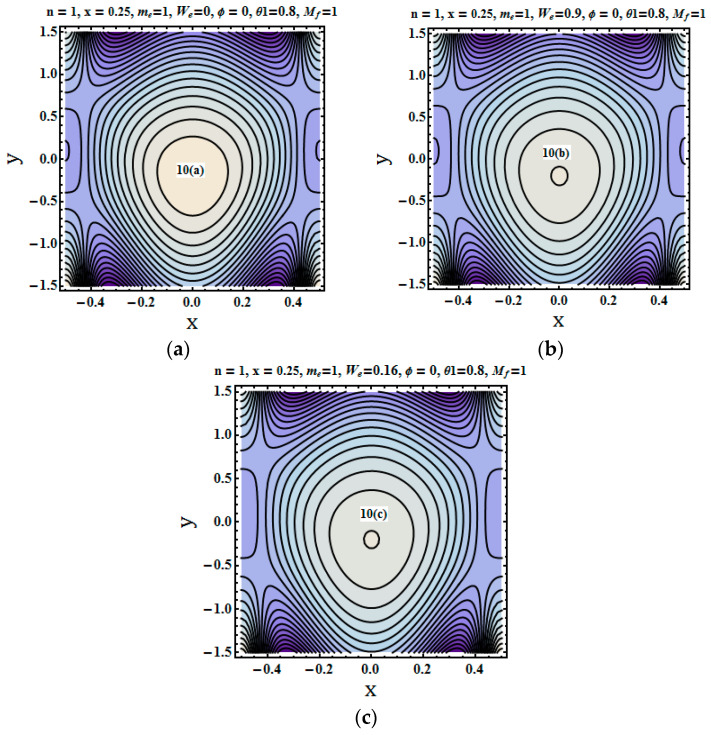
(**a**–**c**) Stream lines for different values of $W_{e}=0,\;W_{e}=0.9,\;W_{e}=0.16$.

Streams lines were plotted to depict the flow pattern. The trapping phenomenon for fluid parameters $M_{f}$ (Hartman number), $m_{e}$ (Hall parameter) and $W_{e}$ (Weissenberg number) was described through plotting the streams lines. Figure 8, Figure 9 and Figure 10 show that the bolus size decreased for increasing values of the Hartman number and Weissenberg number. Figure 10a–c depict the opposite trend that is for higher values of hall parameter bolus size not only increases but number of closed stream lines also increases in count.

### 5.6. Analysis of Entropy Generation Due to Heat Diffusion and Viscous Dissipation

**Figure 11:**
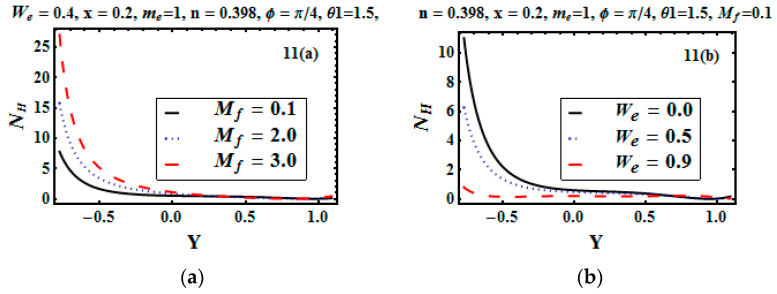
(**a**,**b**) Entropy generation rate due to heat diffusion for different values of $M_{f}$ and $W_{e}$.

**Figure 12:**
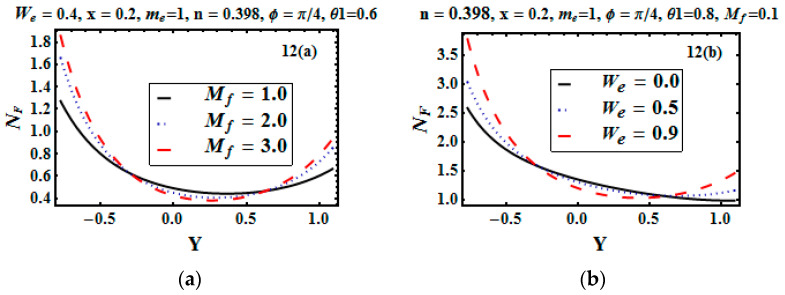
(**a**,**b**) Entropy generation rate due to the viscous dissipation effect for different values of $M_{f}$ and $W_{e}$.

The thermal entropy generation rate and viscous entropy generation are very important in entropy generation. The influence of the Hartman number $\left(M_{f}\right)$ and Weissenberg number $\left(W_{e}\right)$ was studied to configure the entropy generation due to the heat diffusion and viscous dissipation effect. Figure 11a elucidates that for the increasing value of the Hartman number, the heat diffusion rate increased. This physically happens due to the strong magnetic field, which boosts the temperature. Therefore, the diffusion rate increased. Figure 11b illustrates that for higher values of $W_{e},$ the heat diffusion rate decreased. Variation in the entropy generation rate due to viscous dissipation for different values of the Hartman number is observed through Figure 12a,b. The increase in the thermal entropy generation rate at the walls was observed due to resistive forces, while heat production dropped off due to low viscosity at the center of the channel.

## 6. Conclusions

We analyzed the entropy generation via the Ohmic heating and Hall current in peristaltically-flowing Carreau fluid. The conclusions are stated below.

- Entropy generation is not zero at the centerline *y* = 0.
- Heat irreversibility, at the bulk fluid region, is dominant, while at the edges, magnetic and viscous irreversibility dominates.
- The entropy generation profile is parabolic.
- Entropy production boosts for increasing values of the Hartman number and Brinkman number.
- Increasing the value of ξ, which is the temperature difference parameter, reduces both the entropy generation and Bejan number.
- Due to the resistive nature of the magnetic field $B_{0}$, the velocity profile decreases for the Hartman number, while for temperature, it increases.
- The velocity decreases due to the fluid’s Weissenberg number.
- The pressure gradient increases in a wider region for both the Hall parameter and the Hartman number.
- The number of closed circular stream lines encircling the bolus increases with an increase in the values of the Hall parameter.

## References

[1] Hayat T., Khan M.I., Farooq M., Alsaedi A., Waqasas M., Yasmeen T. (2016). Impact of Cattaneo—Christov heat flux model in flow of variable thermal conductivity fluid over a variable thicked surface. Int. J. Heat Mass Transf..

[2] Khan M.I., Waqas M., Hayat T., Alsaedi A. (2017). A comparative study of Casson fluid with homogeneous-heterogeneous reactions. J. Colloid Interface Sci..

[3] Bejan A. (1996). Entropy generation minimization: The new thermodynamics of finite-size devices and finite-time processes. J. Appl. Phys..

[4] Afridi M.I., Qasim M., Makinde O.D. (2019). Entropy generation due to heat and mass transfer in a flow of dissipative elastic fluid through a porous medium. J. Heat Transf..

[5] Afridi M.I., Qasim M., Shafie S. (2017). Entropy generation in hydromagnetic boundary flow under the effects of frictional and Joule heating: Exact solutions. Eur. Phys. J. Plus.

[6] Rashidi M., Bhatti M., Abbas M., Ali M. (2016). Entropy generation on MHD blood flow of nanofluid due to peristaltic waves. Entropy.

[7] Rashidi M.M., Bagheri S., Momoniat E., Freidoonimehr N. (2017). Entropy analysis of convective MHD flow of third grade non-Newtonian fluid over a stretching sheet. Ain Shams Eng. J..

[8] Hayat T., Khan M.I., Qayyum S., Alsaedi A. (2018). Entropy generation in flow with silver and copper nanoparticles. Colloids Surf. A Physicochem. Eng. Asp..

[9] Khan M.I., Qayyum S., Hayat T., Alsaedi A. (2018). Entropy generation minimization and statistical declaration with probable error for skin friction coefficient and Nusselt number. Chin. J. Phys..

[10] Khan M.I., Hayat T., Alsaedi A., Qayyum S., Tamoor S. (2018). Entropy optimization and quartic autocatalysis in MHD chemically reactive stagnation point flow of Sisko nanomaterial. Int. J. Heat Mass Transf..

[11] Wei Y., Wang Z., Qian Y. (2017). A Numerical Study on Entropy Generation in Two-Dimensional Rayleigh-Bénard Convection at Different Prandtl Number. Entropy.

[12] Wang Z., Wei Y., Qian Y.H. (2018). Numerical study on entropy generation in thermal convection with differentially discrete heat boundary conditions. Entropy.

[13] Yang X., He H., Xu J., Wei Y., Zhang H. (2018). Entropy Generation Rates in Two-Dimensional Rayleigh-Taylor Turbulence Mixing. Entropy.

[14] Wei Y., Wang Z., Dou S.H., Qian Y.H. (2017). A novel two-dimensional coupled lattice Boltzmann model for incompressible flow in application of turbulence Rayleigh-Taylor instability. Comput. Fluids.

[15] Wei Y., Wang Z., Dou S.H., Qian Y.H., Yan W.W. (2016). Simulations of natural convection heat transfer in an enclosure at different Rayleigh number using lattice Boltzmann method. Comput. Fluids.

[16] Wei Y., Yang H., Lin Z., Wang Z., Qian Y. (2018). A novel two-dimensional coupled lattice Boltzmann model for thermal incompressible flows. Appl. Math. Comput..

[17] Abbasi F.M., Hayat T., Alsaedi A. (2015). Numerical analysis for MHD peristaltic transport of Carreau–Yasuda fluid in a curved channel with Hall effects. J. Magn. Magn. Mater..

[18] Bhatti M.M., Rashidi M.M. (2017). Study of heat and mass transfer with Joule heating on magnetohydrodynamic (MHD) peristaltic blood flow under the influence of Hall effect. Propuls. Power Res..

[19] Noreen S., Kousar T., Rashid M.M. (2019). Hall, ion slip and ohmic heating effects in thermally active sinusoidal channel. Propuls. Power Res..

[20] Hayat T., Zahir H., Alsaedi A., Ahmad B. (2017). Hall current and Joule heating effects on peristaltic flow of viscous fluid in a rotating channel with convective boundary conditions. Results Phys..

[21] Noreen S., Qasim M. (2015). Influence of Hall Current and Viscous Dissipation on Pressure Driven Flow of Pseudoplastic Fluid with Heat Generation: A Mathematical Study. PLoS ONE.

[22] Rashidi M.M., Yang Z., Bhatti M.M., Abbas M.A. (2018). Heat and mass transfer analysis on MHD blood flow of Casson fluid model due to peristaltic wave. Therm. Sci..

[23] Mekheimer K.S. (2008). Effect of the induced magnetic field on peristaltic flow of a couple stress fluid. Phys. Lett. A.

[24] Noreen S., Malik A., Rashidi M.M. (2019). Peristaltic flow of shear thinning fluid via temperature dependent and thermal conductivity, communication in theoretical physics. Commun. Theor. Phys..

[25] Latham T.W. (1966). Fluid Motions in a Peristaltic Pump. Doctoral Dissertation.

[26] Shapiro A.H., Jaffrin M.Y., Weinberg S.L. (1969). Peristaltic pumping with long wavelengths at low Reynolds number. J. Fluid Mech..

[27] Asghar S., Hussain Q., Hayat T. (2013). Peristaltic flow of reactive viscous fluid with temperature dependent viscosity. Math. Comput. Appl..

[28] Vajravelu K., Sreenadh S., Lakshminarayana P., Sucharitha G., Rashidi M.M. (2016). Peristaltic Flow of Phan-Thien-Tanner Fluid in an Asymmetric Channel with Porous Medium. J. Appl. Fluid Mech..

[29] Hayat T., Noreen S., Alsaedi A. (2012). Effect of an induced magnetic field on peristaltic flow of non-Newtonian fluid in a curved channel. J. Mech. Med. Biol..

[30] Shit G.C., Ranjit N.K., Sinha A., Roy M. (2014). Effect of induced magnetic field on peristaltic transport of a micropolar fluid in the presence of slip velocity. Int. J. Appl. Math. Mech..

[31] Ali N., Hayat T. (2007). Peristaltic motion of a Carreau fluid in an asymmetric channel. Appl. Math. Comput..

[32] Nadeem S., Akram S. (2010). Peristaltic flow of a Williamson fluid in an asymmetric channel. Commun. Nonlinear Sci. Numer. Simul..

[33] Ellahi R., Riaz A., Nadeem S., Ali M. (2012). Peristaltic flow of Carreau fluid in a rectangular duct through a porous medium. Math. Probl. Eng..

[34] Noreen S., Hayat T., Alsaedi A. (2013). Flow of MHD Carreau fluid in a curved channel. Appl. Bionics Biomech..

[35] Hayat T., Yasmin H., Alsaedi A. (2014). Peristaltic motion of Carreau fluid in a channel with convective boundary conditions. Appl. Bionics Biomech..

